# Capgras Syndrome Due to Cannabinoids Use: A Case Report With Radiological Findings

**DOI:** 10.7759/cureus.21412

**Published:** 2022-01-19

**Authors:** Diana Milena Bello Castro, Laura Segura Ayala, Sandra Saavedra, Sandra García, Andrés Felipe Herrera Ortiz

**Affiliations:** 1 Psychiatry, Clinica del Sistema Nervioso Renovar, Bogotá, COL; 2 Psychiatry, Fundación Universitaria Juan N. Corpas, Bogotá, COL; 3 Radiology, Fundación Santa Fe de Bogotá, Bogotá, COL; 4 Radiology, Universidad El Bosque, Bogotá, COL

**Keywords:** affective disorders psychotic, magnetic resonance, marijuana use, cannabis, substance abuse, capgras syndrome

## Abstract

Capgras syndrome is a part of the delusional misidentification syndromes. In this condition, the patient believes that identical individuals have impersonated the people close to them, leading to aggression or even homicide of their relatives. The following article describes the case of a 28-year-old patient with a history of cannabis consumption who arrived at the emergency department due to an unsuccessful murder attempt against his neighbor. At the mental examination, the patient believed their parents were killed some time ago, and impostors were replacing them; laboratory tests showed tetrahydrocannabinol in the urine sample. Therefore, the diagnosis of Capgras syndrome due to cannabis consumption was performed, and treatment was established with two antipsychotics and one mood stabilizer drug, showing satisfactory results after two months.

## Introduction

Capgras syndrome is a part of the delusional misidentification syndromes. In this condition, the patient believes that identical individuals have impersonated the people close to them, leading to aggression or even homicide of their relatives [1-4]. Capgras syndrome was first described in 1923 by Joseph Capgras and Jean Reboul-Lachaux, who reported the case of a 53-year-old female patient, who assured that her children, husband, and later the local police, doctors, neighbors, and even herself were being replaced by doubles [5]. The prevalence of Capgras syndrome among the general population is 0.12%, while in the psychiatric population rises to 1.3% [6-8]. The etiology of Capgras syndrome can be variable, being in most of the cases associated with preexistence neuropsychiatric conditions [4].

Even though Capgras syndrome has been reported in close to 258 individuals, its relationship with recreational drugs represents an infrequent trigger described in only seven cases in the literature [4]. This paper aims to report a case of a young male patient without a previous history of psychiatric illnesses who debuted with a psychotic episode associated with Capgras syndrome in which the only identifiable etiology was cannabis intoxication.

## Case presentation

A 28-year-old male patient with no records of family psychiatric illnesses, but with a history of cannabis consumption for eight years and hetero-aggressive behaviors toward his parents for the last three months, was brought by the police to a psychiatric hospital due to an unsuccessful murder attempt against his neighbor. At the initial evaluation, a patient with motor anxiety, neglect of his care, hyporexia, and decreased necessity of sleep was evident.

The mental status examination showed an aware patient, oriented to time, place, and person, seductive, with expansive mood, illogical thoughts, delusional ideas of the paranoid and megalomaniac spectrum, associated with poorly modulated affect, null introspection, and compromised judgment; no hallucinatory attitude manifested. The patient identified himself as a famous musician and his grandfather as a renowned terrorist. During the mental examination, it was evidenced that the patient believed their parents were killed some time ago, and impostors were now replacing them; therefore, he desired to murder “the impostors”.

**Figure 1:**
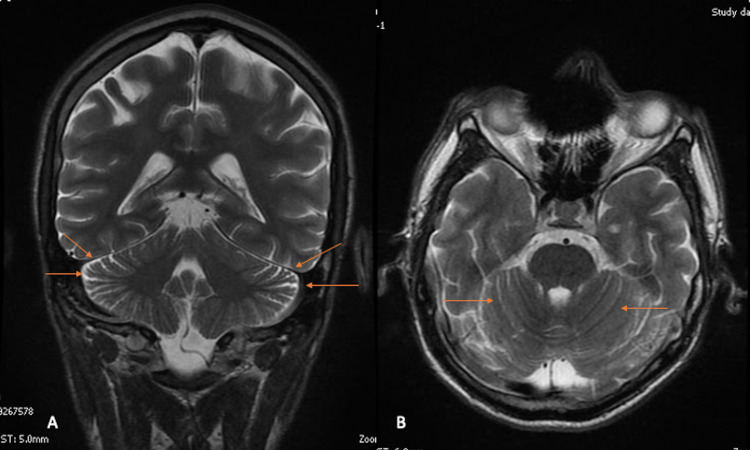
T2-weighted brain MRI in coronal view (A) and axial view (B) showing (arrows) a slight accentuation of cerebellar folia in the context of a patient with Capgras syndrome. MRI: magnetic resonance imaging

Some laboratory tests were requested to rule out other secondary delusional disorders, showing a cannabinoid screening test in urine with positive results for tetrahydrocannabinol, no human immunodeficiency virus (HIV) infection, and no syphilis. Also, hemogram, urine sample, and creatinine levels were within normal parameters. A brain magnetic resonance imaging (MRI) was conducted to rule out structural lesions, showing a slight accentuation of cerebellar folia (Figure 1).

Due to the clinical presentation of this patient, exhibiting paranoid and megalomaniac delusional ideas, in addition to the perception of impostors, and the results of the laboratory tests, which showed persistence of tetrahydrocannabinol in the urine sample, the final diagnosis was set as a Capgras syndrome possibly caused by cannabis consumption.

Once the diagnosis was performed, the treatment was started with lorazepam 1 mg/day, valproic acid syrup 250 mg/5 ml every 12 hours, clozapine 100 mg in the morning, and 300 mg at night, clonidine 150 mcg every 12 hours, and pipotiazine 25 mg intramuscular injection once a month. One month after the hospital admission, the patient persisted with psychotic symptoms with a high risk of parricide due to Capgras syndrome. Therefore, we decided to remove lorazepam and clonidine, leaving the patient with valproic acid syrup 750 mg/5 ml every 12 hours, clozapine 200 mg in the morning, and 300 mg at night, in addition to pipotiazine. One month later, the patient presented a complete resolution of the Capgras syndrome; therefore, discharge was given with an ambulatory control appointment with psychology and psychiatry. A detailed timeline is shown in Table 1.

**Table 1:** Patient timeline for relevant past medical history and interventions, including relevant personal, family, and psychosocial history; important past interventions; outcomes ELISA: enzyme-linked immunosorbent assay; HIV: human immunodeficiency virus; MRI: magnetic resonance imaging; VDRL: Venereal Disease Research Laboratory

Relevant Past Medical History and Interventions (Cannabis Consumption in the Past Eight Years)				
Date	Summaries from Initial and Follow-up Visits	Diagnostic Testing (Including Dates)	Interventions	
08/09/2021	The patient arrived with hetero-aggressive behaviors toward his parents for the last three months. During the mental examination, it was evidenced that the patient believed their parents were killed time ago, and impostors were now replacing them; therefore, he desired to murder “the impostors”.	Cannabinoid screening test in urine with positive results for tetrahydrocannabinol (10/09/2021). Hemogram, urine sample, creatinine, VDRL, and HIV ELISA were within normal parameters (10/09/2021). A brain MRI was conducted to rule out structural lesions, showing a slight accentuation of cerebellar folia (11/09/2021).	Lorazepam 1 mg/day, valproic acid syrup 250 mg/5 ml every 12 hours, clozapine 100 mg in the morning, and 300 mg at night, clonidine 150 mcg/12 hours, and pipotiazine 25 mg intramuscular injection once a month	
08/10/2021	The patient persisted with psychotic symptoms with a high risk of parricide due to Capgras syndrome	No investigations performed	We decided to remove lorazepam and clonidine, leaving the patient with valproic acid syrup 750 mg/5 ml every 12 hours, clozapine 200 mg in the morning, and 300 mg at night, in addition to pipotiazine	
08/11/2021	The patient presented a complete resolution of all psychiatric symptoms	No investigations performed	Discharge was given with an ambulatory control appointment with psychology and psychiatry	

## Discussion

Capgras syndrome associated with cannabis has been described mainly in male patients between 17 and 30 years old, either after chronic or acute cannabinoid consumption, which is consistent with the information described in this case [1,9].

The etiology of Capgras syndrome can be divided into organic (43%), functional (56%), mixed (<1%), and unspecified (<1%) [4]. Those classified in the organic group are mainly associated with other conditions such as organic delusional disorders, dementia, delirium, and drug abuse [4]. Several illegal substances that precipitate Capgras syndrome can be identified within the drug abuse group, such as cocaine, alcohol, and cannabis [1,10,11].

Drug abuse represents an extremely rare precipitating factor for Capgras syndrome; therefore, it is mandatory to rule out first other organic and functional conditions that may trigger the disease, as was done in this scenario by requesting the Venereal Disease Research Laboratory (VDRL), HIV tests, and MRI [4]. The main advantage of imaging modalities such as MRI is the capacity to exclude structural or vascular etiologies that may be precipitating Capgras syndrome.

The symptoms associated with Capgras syndrome are usually paranoid delusions (35%), dissociation (3%), affective symptoms (52%), aggression (32%), homicide (4%), auditory hallucinations (25%), visual hallucinations (13%), and other misidentification syndromes (8%) [4]. Our patient only manifested paranoid delusions, affective symptoms, and aggression, representing the typical clinical picture.

Another interesting aspect is that in most of the Capgras syndrome cases associated with cannabis, the misidentified subjects were the patient’s parents, which coincides with the clinical presentation reported in this case [1,9]. We consider that the reason for this may be the fact that delusions are elaborated based on the patient’s experiences and are mainly reflected over individuals with the closest bond with the patient. Given the age group of individuals who consume cannabis, the family nucleus is usually constituted by their parents, making them the individuals with the closest bond to the patient, which explains why the parents are the object of the delusion [4].

It has been published previously that the clinical presentation of Capgras syndrome may vary according to the etiology. For example, functional Capgras are more prone to present auditory hallucinations, aggression, homicide, and other misidentification syndromes, while organic Capgras show more visual hallucinations [4]. However, even though our patient presented an organic Capgras due to substance abuse, the patient did not manifest any hallucinations.

Radiological findings of Capgras syndrome have been poorly reported in the literature; however, some authors have described the presence of T2-weighted-fluid-attenuated inversion recovery (FLAIR) hyperintensities located in the frontal, occipital, and temporal subcortical white matter and semi-oval circle, in addition to global brain atrophy [4,12,13]. In this case, our patient showed a slight accentuation of cerebellar folia, which is an imaging pattern that has not been described previously in the literature. We do consider that the accentuation of the cerebellar folia may represent a certain degree of cerebellum atrophy.

In this case, our patient presented a complete resolution of symptoms after two months of treatment with a combination of two antipsychotics and one mood stabilizer drug. However, the late recovery of this case and the pharmacological treatment established have not been reported previously in patients with Capgras. Most cases describe an improvement after two weeks of treatment with only first-line antipsychotics [1,9].

Although our patient’s response time to antipsychotic treatment differs from the other reported cases, it is within the expected period of action. However, the variability in response time can be attributed to pharmacodynamic and pharmacokinetic causes, determined by genetic, environmental, or disease-specific factors that are independent in each patient [14].

## Conclusions

The association between Capgras syndrome and cannabis consumption represents an extremely rare condition; therefore, it is mandatory first to rule out other organic and functional diseases associated with Capgras syndrome. In this case, cannabis was identified as a likely trigger for Capgras and not a definite cause for this presentation. The combination of two antipsychotics and one mood stabilizer drug showed to be an effective treatment for Capgras syndrome in this scenario. We consider that the radiological findings associated with Capgras have been poorly described in the literature, making it a potential topic for further research.
